# Supplementary material for: The Dual Prey-Inactivation Strategy of Spiders—In-Depth Venomic Analysis of Cupiennius salei
Source: Toxins (Basel). 2019 Mar 19;11(3):167. doi: 10.3390/toxins11030167 (PMC6468893; doi:10.3390/toxins11030167)
Supplement: Supplementary file 1 [file toxins-11-00167-s001.zip › Supplementary Fig S1.1-S1.19/Supplementary Fig S1.15.pdf]

Supplementary Fig S1.15

CLUSTAL O(1.2.4) multiple sequence alignment: Kunitz domain-containing proteins

```
KCP_1_CUPSA                     --------MLMVFLLASSIGVALSSPFV----RPNYNNIPQCLQPMESGLCLAYIPSFYY
KCP_2_CUPSA                     --------MLMVFLLASSIGVALSSPFV----RPNYNNIPQCLQPMESGLCLAYIPSFYY
KCP_ALOMA                       --------MVMFLLFASSIGLALSNPLV---SQPNYDNIPQCLQPPVSGMCLAYFPSYYY
KCP_VIRFA                       --------MLMIFVLGATIGIAFSTPVQFGDIRPNYDNTPQCMQPSDGGMCLAYFPSFYY
tr_A0A087UV99_STEMI             ----MTNRIVQAVVVISAIGIVFSATIQ-PYVPPSYENIPQCLQPAESGLCLAYFLSYYY
sp_W4VSH9VKT1_TRILO             ------MNFELIYVSSLLLGICLANQAD---V-----VPSDCNLPADAGMCYAYFPMFFY
                                        :    :    :*: ::                :*  *  .*:* **:  ::*

KCP_1_CUPSA                     NAAKGTCDNFVYGGCQGNSNRFFSFDECMAKCGNFKPETNENFKLVLENGEDQDSSEDSD
KCP_2_CUPSA                     NAAKGTCDNFVYGGCQGNSNRFFSFDECMAKCGNFKPETNENFKLVLENGEDQDSSEDSG
KCP_ALOMA                       NPSKETCDTFIYGGCQGNANRFFSFDECMAKCGGFKQEPNDSFKLVLENGEDQNPS-ASE
KCP_VIRFA                       NPAKKTCESFIYGGCQGNSNRFFSFDECMSTCGGFEPELN-EFKLVLESDEDEDTSV---
tr_A0A087UV99_STEMI             NPSTRTCETFVYGGCQGNANRFNTFEQCMATCNGTSATTI-HFELITES-------NPSE
sp_W4VSH9VKT1_TRILO             DASSRKCLNFIYGGCGGNANRFWSEAECMEKCGGGGGGGGS-------SDGSQKTAKMLN
                                : :. .* .*:**** **:*** :  :** .*..              .

KCP_1_CUPSA                     QKVDDICSQPSVMGPCRAMMPRFYFNGD--KCQSFIFGGCEGNGNNFRSVEECEQRCGSN
KCP_2_CUPSA                     QQVDAICSLPSVMGPCRAMMPRFYFNGD--KCQSFIFGGCEGNGNNFRSVEECEQRCGSN
KCP_ALOMA                       QQVEDICSLSTEMGPCRAMMPRFYFNGD--KCESFIYGGCEGNGNNFKTLEECQQRCGST
KCP_VIRFA                       DESGAICSLHSETGPCKAMMPRFYFNGN--KCESFIFGGCKGNKNNFLSVEECEQKCGPK
tr_A0A087UV99_STEMI             KEVQDPCTLPVESGQCRAAIPRFYFNGE--KCENFIYGGCDGNANNFRTIEDCEKTCAPS
sp_W4VSH9VKT1_TRILO             LDLGDICSLEKKVGPCKAHMPRYYFNRETGLCEEFIYGGCSGNHNNFQTKEQCESFCAPG
                                 .    *:     * *:* :**:*** :   *:.**:***.** *** : *:*:. *.

KCP_1_CUPSA                     STEEVGENSDEASEKVSKEESSEEGSDENSEESKN
KCP_2_CUPSA                     STEEVGENSDEASEKVSKEESSEEGSDENSEESKN
KCP_ALOMA                       SSSEEEADNSEETEKASEEAVAEEASVERSEDS--
KCP_VIRFA                       TSEEVDASSENKSD---------------------
tr_A0A087UV99_STEMI             FSEQSGEQPIQ------------------------
sp_W4VSH9VKT1_TRILO             NSPRPEEETRKRTKQSY------------------
                                 : .   .  :
```

Percent Identity Matrix - created by Clustal2.1

1:	KCP_1_CUPSA	100.00	98.01	69.19	65.34	50.30	36.26
2:	KCP_2_CUPSA	98.01	100.00	69.70	66.48	50.30	36.26
3:	KCP_ALOMA	69.19	69.70	100.00	61.36	53.25	41.52
4:	KCP_VIRFA	65.34	66.48	61.36	100.00	49.70	43.64
5:	tr_A0A087UV99_STEMI	50.30	50.30	53.25	49.70	100.00	41.25
6:	sp_W4VSH9VKT1_TRILO	36.26	36.26	41.52	43.64	41.25	100.00

Proteomic coverage trypsin

>KCP_1_CUPSA kunitz domain-containing protein isoform 1

MLMVFLLASS IGVALS**SPFV RPNYNNIPQC LQPMESGLCL AYIPSFYYNA AKGTCDNFVY GGCQGNSNRF FSFDECMAKC**
GNFKPETNEN FKLVLENGED QDSSEDSDQK VDDICSQPSV MGPCRAMMPR **FYFNGDKCQS FIFGGCEGNG NNFR**SVEECE
QR**CGSNSTEE VGENSDEASE KVSK**EESSEE GSDENSEESK N

>KCP_2_CUPSA kunitz domain-containing protein isoform 2

MLMVFLLASS IGVALS**SPFV RPNYNNIPQC LQPMESGLCL AYIPSFYYNA AKGTCDNFVY GGCQGNSNRF FSFDECMAKC**
GNFKPETNEN FKLVLENGED QDSSEDSGQQ VDAICSLPSV MGPCRAMMPR **FYFNGDKCQS FIFGGCEGNG NNFR**SVEECE
QR**CGSNSTEE VGENSDEASE KVSK**EESSEE GSDENSEESK N

Proteomic coverage chymotrypsin

>KCP_1_CUPSA kunitz domain-containing protein isoform 1

MLMVFLLASS IGVALSSPFV RPNYNNIPQC LQPMESGLCL AYIPSFY**YNA AKGTCDNFVY GGCQGNSNRF FSFDECMAKC GNFKPETNEN FKLVLENGED QDSSEDSDQK VDDICSQPSV MGPC**RAMMPR FYFNGDKCQS F**IFGGCEGNG NNF**RSVEECE QRCGSNSTEE VGENSDEASE KVSKEESSEE GSDENSEESK N

>KCP_2_CUPSA kunitz domain-containing protein isoform 2

MLMVFLLASS IGVALSSPFV RPNYNNIPQC LQPMESGLCL AYIPSFY**YNA AKGTCDNFVY GGCQGNSNRF FSFDECMAKC GNFKPETNEN F**KLVLENGED QDSSEDS**G**Q**Q** VD**A**ICS**L**PSV MGPCRAMMPR FYFNGDKCQS F**IFGGCEGNG NNF**RSVEECE QRCGSNSTEE VGENSDEASE KVSKEESSEE GSDENSEESK N

Supplementary Fig S1.15 Multiple sequence alignment of KCP from the venom glands of *C. salei* (KCP_1_CUPSA [MH766640]; KCP_2_CUPSA [MH766641]), *A. marikovskyi* (KCP_ALOMA), *V. fasciatus* (KCP_VIRFA), *S. mimosarum* (tr_A0A087UV99_STEMI), and *T. loki* (sp_W4VSH9VKT1_TRILO). Signal peptides are marked in black characters, cysteines in white with a black box, positively charged amino acids in pink, negatively charged amino acids in blue, hydrophobic amino acids in red and polar amino acids in green. Possible serine protease interaction sites are highlighted in yellow.
